# Exploring diurnal variation using piecewise linear splines: an example using blood pressure

**DOI:** 10.1186/s12982-017-0055-5

**Published:** 2017-02-02

**Authors:** Jamie M. Madden, Xia Li, Patricia M. Kearney, Kate Tilling, Anthony P. Fitzgerald

**Affiliations:** 1 RCSI Population and Health Sciences, Royal College of Surgeons in Ireland, Dublin, Ireland; 2 Department of Mathematics and Statistics, La Trobe University, Melbourne, Australia; 3 Department of Epidemiology and Public Health, University College Cork, Cork, Ireland; 4 School of Social and Community Medicine, University of Bristol, Bristol, UK; 5 Department of Statistics, University College Cork, Cork, Ireland

**Keywords:** Circadian modeling, Mixed-effects models, Biostatistics, Blood pressure variability, Blood pressure patterns

## Abstract

**Background:**

There are many examples of physiological processes that follow a circadian cycle and researchers are interested in alternative methods to illustrate and quantify this diurnal variation. Circadian blood pressure (BP) deserves additional attention given uncertainty relating to the prognostic significance of BP variability in relation to cardiovascular disease. However, the majority of studies exploring variability in ambulatory blood pressure monitoring (ABPM) collapse the data into single readings ignoring the temporal nature of the data. Advanced statistical techniques are required to explore complete variation over 24 h.

**Methods:**

We use piecewise linear splines in a mixed-effects model with a constraint to ensure periodicity as a novel application for modelling daily blood pressure. Data from the Mitchelstown Study, a cross-sectional study of Irish adults aged 47–73 years (n = 2047) was utilized. A subsample (1207) underwent 24-h ABPM. We compared patterns between those with and without evidence of subclinical target organ damage (microalbuminuria).

**Results:**

We were able to quantify the steepest rise and fall in SBP, which occurred just after waking (2.23 mmHg/30 min) and immediately after falling asleep (−1.93 mmHg/30 min) respectively. The variation about an individual’s trajectory over 24 h was 12.3 mmHg (standard deviation). On average those with microalbuminuria were found to have significantly higher SBP (7.6 mmHg, 95% CI 5.0–10.1) after adjustment for age, sex and BMI. Including an interaction term between each linear spline and microalbuminuria did not improve model fit.

**Conclusion:**

We have introduced a practical method for the analysis of ABPM where we can determine the rate of increase or decrease for different periods of the day. This may be particularly useful in examining chronotherapy effects of antihypertensive medication. It offers new measures of short-term BP variability as we can quantify the variation about an individual’s trajectory but also allows examination of the variation in slopes between individuals (random-effects).

**Electronic supplementary material:**

The online version of this article (doi:10.1186/s12982-017-0055-5) contains supplementary material, which is available to authorized users.

## Background

There are many examples of physiological processes that follow a circadian cycle such as cortisol, intraocular pressure and body temperature where abnormalities in these patterns have been shown to be related to depression [1], glaucoma [2] and delayed sleep-phase disorder [3]. The ability to analyse and capture features of these cycles remains a challenge but is necessary to get a deeper understanding of the mechanisms behind them. For example, the cardiovascular system shows clear circadian rhythmicity where researchers are interested in alternative methods to illustrate and quantify this diurnal variation [4]. Circadian blood pressure (BP) represents a situation where diurnal variation deserves additional attention given the uncertainty relating to the prognostic significance of BP variability (BPV) [5–8]. The benefits of using ambulatory blood pressure monitors (ABPM) in addition to clinic measurements in the diagnosis and management of hypertension are well established [9, 10]. As well as mean day, night and dip values, ABPM provides measures of short-term BPV and individual profile patterns. The majority of studies examining short-term BPV have focused on summary measures such as the standard deviation (SD) of ABPM readings over the day. These summary measures are easily obtained without the need for advanced statistical techniques [5–8, 11, 12] but ignore the temporal nature of the data. To date relatively little work has modelled 24 h ABPM profiles to exploit the full potential of ABPM data to capture short-term BPV [13]. Moreover, there are a lack of studies exploring circadian patterns and specifically, studies examining differences in patterns among different groups of individuals.

Cosinor analysis which incorporates a sinusoidal function has been the most common approach to modelling 24 h blood pressure (BP) [14–17], while a similar method, Fourier analysis [18, 19], has also been implemented. These approaches have focused on between-group effects (fixed-effects) where typically inferences are based on estimated differences in model parameters between particular groups of patients, such as comparing the estimated amplitude or midline estimating statistic of rhythm (MESOR) between groups of individuals on different antihypertensive agents obtained in cosinor analysis [16]. The focus of fixed-effects is on population trajectories. However one of the main advantages of ABPM is that we obtain individual BP profiles and modelling subject-specific trajectories involves incorporating subject-specific effects (random-effects). To model mean profiles Selwyn and Difranco [20] used a hierarchical model incorporating a 4th degree polynomial. Lambert et al. extended on this by incorporating restricted cubic splines to model the mean BP profiles [13]. More recently, Edwards and Simpson [21] utilised orthonormal polynomials in a linear mixed model in a group of hypertensive subjects. Both polynomials and cubic splines, by their nature, have the ability to produce well-fitting curves to the data but have the disadvantage that the corresponding coefficients are challenging to interpret directly.

As an alternative we propose using piecewise linear splines in a mixed-effects model as a different approach for modelling ABPM data. Although linear splines have been used to model BP change over years and gestational age [22], using them to explore daily patterns of BP represents a novel method for analysing ABPM. This approach has the advantage that coefficients represent something meaningful, in this case the slope of BP at different periods of the day. To date it is unclear if different underlying circadian BP patterns exist across various groups of the population. This method allows slopes at a group level (and individual level) to be easily compared. Furthermore, using random-effects we want to predict and plot curves at an individual level and to explore BPV within each period of the day. Thus the aim of this study is twofold (1) to introduce and describe a mixed-effects piecewise linear model in relation to BP; (2) to apply our method to a middle-aged population sample and explore their circadian BP patterns. We also introduce and present a constraint for our model that ensures periodicity, so that on the average BP is the same 24 h later. We are particularly interested in identifying distinct differences in the shape of mean curves at a group level. For purposes of illustration of the models at a group level, we will compare those with and without evidence of subclinical target organ damage (TOD), specifically microalbuminuria.

## Methods

### Study population

The analysis utilises data from the Mitchelstown Study, a cross sectional study of middle-aged men and women, recruited in Ireland 2010–2011. A description of the study design is available from previous publications [23, 24]. The study recruited patients attending a single large primary care centre, the LivingHealth Clinic, in Mitchelstown. Participants completed a detailed health and lifestyle survey questionnaire, and attended for a physical examination including height, weight, blood pressure, fasting blood samples and urine samples. ABPM was offered to all participants. All participants provided written informed consent and ethical approval was obtained from the Research Ethics Committee of the Cork Teaching Hospitals.

### Blood pressure measurements

Study BP was measured three times after 5 min of rest in a seated position by experienced research nurses using an OMRON M7 blood pressure monitor (OMRON Healthcare, The Netherlands). The average of the second and third measurements was used for analyses. Ambulatory BP was measured using dabl ABPM system (dabl ltd., Ireland) with the Meditech ABOM-05 Monitor (Meditech LTD., Hungary). The monitors were programmed to obtain readings every 30 min and remained in place for 24-h. Participants kept diaries of wake and sleep periods, which were used to calculate sleep and waking times. Only participants with a minimum of 20 measurements during the day and a minimum of 7 measurements during the night period were included in the analysis. Additionally, any participants with data lacking for more than two consecutive hourly intervals were excluded [25].

### Target organ damage

Each participant provided an early-morning spot urine sample on the day of their appointment. Laboratory analyses included analysis for albumin:creatinine ratio (ACR). Microalbuminuria is defined as ACR ≥1.1 mg mmol^−1^ [26].

### Statistical analysis

#### Linear mixed model: linear splines

The linear mixed model [27, 28] is a well-recognised tool in the analysis of longitudinal data and its ability to obtain both population (fixed-effects) and subject-specific (random-effects) trajectories makes it particularly appealing for the analysis of ABPM data. However, its use to-date has focused on BP following a smooth curvature trajectory which results in spline and polynomial coefficients that are of no direct clinical relevance. Piecewise linear functions or linear splines offer an alternative. These involve segregating the data into different segments across time initially assuming the segments are the same for everyone. Within each partition, a linear spline is fitted and where these are connected are known as knot points. The corresponding coefficient of each spline represents the rate of increase or decrease of BP during each time period.

#### Knot selection

The position of the knot points were determined based on a number of factors. Firstly, to get a general sense of the shape of the data and determine regions of interest (how many knots were required), we plotted an average curve of BP including all participants to determine common knot points. In addition we incorporated prior known characteristics of BP. The period of awakening corresponds with an abrupt and steep acceleration of BP and for many the maximum value obtained during this morning period corresponds to their maximum BP reached throughout the day [29]. We also know that BP gradually falls throughout the day and usually dips to its lowest value during the sleeping period [9]. Since waking and sleeping times are clearly important in terms of changes in BP we decided that the use of these times as two additional subject-specific knot points was appropriate. We were able to create these subject-specific knots using the wake and sleep times reported by the participant. We had 49 readings for each individual, where the first reading was t_1_ (12 p.m.) and the final reading was t_49_ (12 p.m. the following day). Individual waking and sleeping times were included within this range (t_1_ − t_49_). For each individual we created n linear splines, where the kth spline:

1 $$\begin{array}{*{20}l} {{\text{s}}_{\text{k}}\,\left( {\text{t}} \right) \, = \, 0 \, } \hfill & \quad{{\text{if}}\quad {\text{t}} \le {\text{ t}}_{\text{ki}} } \hfill \\ {{\text{s}}_{\text{k}}\,\left( {\text{t}} \right) \, = {\text{ t}}_{\text{i}} - {\text{ t}}_{\text{ki}} } \hfill &\quad {{\text{if}}\quad {\text{t }} < {\text{t}}_{\text{k}} \le {\text{ t}}_{{{\text{ki}} + 1}} \quad {\text{for}}\;{\text{k}} = 1, \ldots ,{\text{n}}} \hfill \\ {{\text{s}}_{\text{k}}\,\left( {\text{t}} \right) \, = {\text{ t}}_{{{\text{ki}} + 1}} - {\text{ t}}_{\text{ki}} } \hfill & \quad{{\text{if}}\quad {\text{t}}_{\text{i}} > {\text{ t}}_{{{\text{ki}} + 1}} } \hfill \\ \end{array}$$

Incorporating these linear splines into a linear mixed effects model for BP we get:

2 $$\begin{aligned} & BP_{ij} = (\beta_{0} + b_{0i} ) + \sum\limits_{k = 1}^{n} {(\beta_{k} + b_{ki} )} s_{k} + \varepsilon_{ij} \\ & b_{i} \sim\;MVN\left( {0,\sum_{b} } \right),\quad \varepsilon_{ij} \sim\;N\left( {0,\sum_{\varepsilon } } \right) \\ \end{aligned}$$

where *BP*_*ij*_ is the BP value for the *jth* measurement on the *ith* person, at time *t*_*ij*_, the *β*'*s* are the fixed effects coefficients associated with the average intercept at *β*_0_ (BP at 12 p.m.) and the average slopes (*β*_*k*_’*s*) between knot points, *b’*s are the random effects associated with the average intercept (*b*_0*i*_) and average slopes between knot points, and *ε*_*ij*_ representing the individual-level residuals from the model. The model is extended by incorporating the subject-specific knots in the *s*_*k*_ term. It is assumed the random effects (*b*_*i*_) have zero mean and an unstructured variance–covariance matrix Σ_*b*_. The individual level residuals have mean zero and variance–covariance matrix Σ_ε_.

To expand Eq. (2) to include the restriction that on the average BP is the same 24 h later we define an equation which states average, subject-specific change in BP over 24 h is zero:

3 $$\sum\limits_{k = 1}^{n} {w_{ki} } \beta_{ki} = 0$$

where *w*_*ki*_ is the width of the *kth* interval (and where those involving wake and sleep times are subject-specific width intervals). Rewriting this in terms of *β*_1_ gives:

4 $$\beta_{1i} = - \frac{{\mathop \sum \nolimits_{k = 2}^{n} w_{ki} \beta_{ki} }}{{w_{1i} }}$$

which implies:

5 $$\mathop \sum \limits_{k = 1}^{n} \beta_{ki} s_{k} = \beta_{1i} s_{1} + \mathop \sum \limits_{k = 2}^{n} \beta_{ki} s_{ki} = \mathop \sum \limits_{k = 2}^{n} \beta_{ki} s_{ki}^{*}$$

where $s_{ki}^{*} = s_{ki} - \frac{{w_{ki} }}{{w_{1i} }}s_{1i} ,$ which allows us rewrite (2) as

6 $$BP_{ij} = \left( {\beta_{0} + b_{0i} } \right) + \mathop \sum \limits_{k = 2}^{n} \left( {\beta_{k} + b_{ki} } \right)s_{ki}^{*} + \varepsilon_{ij}$$

To explore a group effect the model can easily incorporate a variable of interest, in this case TOD (microalbuminuria), as a dichotomous covariate. We further extended the model allowing the shape of the trajectory to depend on TOD by including interactions between TOD and each linear spline slope. Comparing this model with one without any interactions allowed us to test if the overall trajectory of BP was different between the two groups across the day. Additionally we were able to test if slopes between the groups differed at specific locations throughout the day. We adjusted for confounders by adding them into the model as fixed effects. In additional models we tested the effect of allowing the residual variance to differ between those with and without microalbuminuria. Similarly we tested the impact of allowing the interaction terms of microalbuminuria with each linear spline to be random to determine if there was heterogeneity of variance between the groups at any period of the day. Although we used an unstructured covariance structure for our models, we assumed these interaction terms to be independent of the other random-effects parameters. These interactions represented the difference in variation between the microalbuminuria groups within each segment. For all models explored we allowed all the linear spline terms to be random.

As individual ABPM readings taken close in time are likely to be correlated, a model with an independent residual correlation structure may not be appropriate. We compared this to a model with a first-order autoregressive AR(1) structure and examined a plot of the auto-correlation function (ACF) to detect violations of the assumption of independence. Allowing for temporal correlation can potentially result in a large improvement in the precision of parameter estimates [30].

Models were compared formally by a likelihood ratio test (LRT) [28, 31]. The appropriate variance and residual function structures were also identified using a LRT in addition with an ACF plot. R-squared (*R* ^2^) statistic is often presented as a summary measure for linear models but due to theoretical or practical problems is rarely presented for mixed-models. Nakagawa and Schielzeth discuss these issues and present a general but simple method for calculating an appropriate *R* ^2^ for random intercept mixed-models [32]. Johnson extended this to include random slope models which we implement in our analysis [33].

The parameters for our final models were estimated using restricted maximum likelihood estimation (REML) as this method produces unbiased estimates unlike maximum likelihood (ML) estimation [30]. Subject-specific trajectories were estimated using Empirical Best Linear Unbiased Predictors (EBLUPs) of the random-effects [28]. Residual diagnostic plots were examined to verify model assumptions including normality of both random-effects and residual errors and that the error terms had constant variance. In addition a visual predictive check (VPC) was performed in which the estimated mean and the 90% prediction interval from our model were plotted together with the observed BP values and the 90% interquantile range of the observations. The purpose of the VPC is to assess graphically if predictions from the fitted model reproduce the central trend and variability of BP in the observed data, when plotted against time. It is an internal validation method that assesses the goodness-of-fit [34]. All analysis was completed for both SBP and DBP. All analysis were implemented in R [35] and parameter estimation for the mixed-effect model was carried out by means of the lme command in nlme package [36]. Sample R code is provided to ensure reproducibility and improve dissemination of methods (see Additional file 1).

#### Validation

Although difficult to interpret the coefficients, polynomial regression can still be a useful tool in the analysis of medical data to plot the trajectory of non-linear or curvilinear relationships [37]. As a method of validation for our approach we additionally implemented a linear mixed model with orthogonal polynomials across time in both the fixed and random effects, similar to that of Edwards and Simpson [21]. We wanted to determine if linear splines were capable of capturing the circadian rhythm of BP. This was investigated by comparing the trajectories obtained from both methods to determine if they followed similar patterns. A similar process to that of the piecewise model was followed when fitting the polynomial model. As we were only concerned to know if the general shape could be captured by our piecewise approach we were not worried about over-fitting the polynomial model. We implemented a model up to a 6th order polynomial allowing all the terms to be random.

## Results

Of 3051 individuals invited to participate, 2047 (response rate: 67%) completed the questionnaire and physical examination component. ABPM was offered to all 2047 participants and it was completed by 1207 (response rate: 58%) people, of whom 1008 had a minimum of 20 day and 7 night measurements respectively. Of these 886 had no data missing for more than two consecutive hourly intervals, and the main clinical characteristics of these participants are presented in Table 1. Overall, participants had a mean age of 59.9 (5.5) and the majority were female (55%). Sixty percent were classified as hypertensive. Also presented in Table 1 are the characteristics of the full sample which shows the ABPM sub-sample follows a similar distribution in terms of age, sex, BMI and the presence of microalbuminuria. However, the proportion of those with hypertension was higher amongst those in ABPM sub group than in overall study population (60 vs 47%).

**Table 1:** Baseline characteristics

Characteristic	Total (n = 2047)	ABPM (sub-sample)
		Total (n = 886)
Age, years	59.8 (5.5)	59.9 (5.5)
Gender, male n (%)	1008 (49.2)	401 (45.3)
*BMI, n (%)*		
Underweight/normal (<25 kg/m^2^)	447 (21.9)	195 (22.0)
Overweight (25–30 kg/m^2^)	925 (45.3)	380 (42.9)
Obese (≥30 kg/m^2^)	668 (32.8)	310 (35.0)
Office SBP, mmHg	129.6 (16.9)	134.7 (17.7)
Office DBP, mmHg	80.1 (9.8)	83.1 (10.2)
Hypertension, n (%)	951 (46.5)	528 (59.7)
Microalbuminuria	215 (10.6)	62 (7.0)

Data are mean (SD). *BMI*:body mass index, *ABPM* ambulatory blood pressure monitor. Hypertension: ≥140/90 mmHg and/or on antihypertensive treatment

**Fig. 1:**
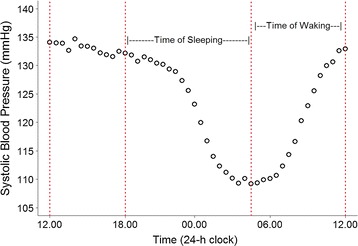
Plot of average SBP over 24 h which helped identify 6 p.m. and 4 a.m. as common knot points for all participants where there was a notable change in trajectory of BP. Also highlighted are the periods where individuals woke and went to sleep. In addition to the two common points, we were able to obtain additional (two) subject-specific knot points at wake and sleep times

The plot of average SBP for the 886 subjects is presented in Fig. 1. Based on this plot we identified two common knot points where the trajectory of SBP changed notably at 6 p.m. and 4 a.m. In addition to these two points we were able to include two subject-specific knot points for each participant based on the time an individual woke and went to sleep (Fig. 1). This meant each participant was assigned 4 knot points which in turn resulted in their SBP pattern being broken into 5 linear segments.

**Fig. 2:**
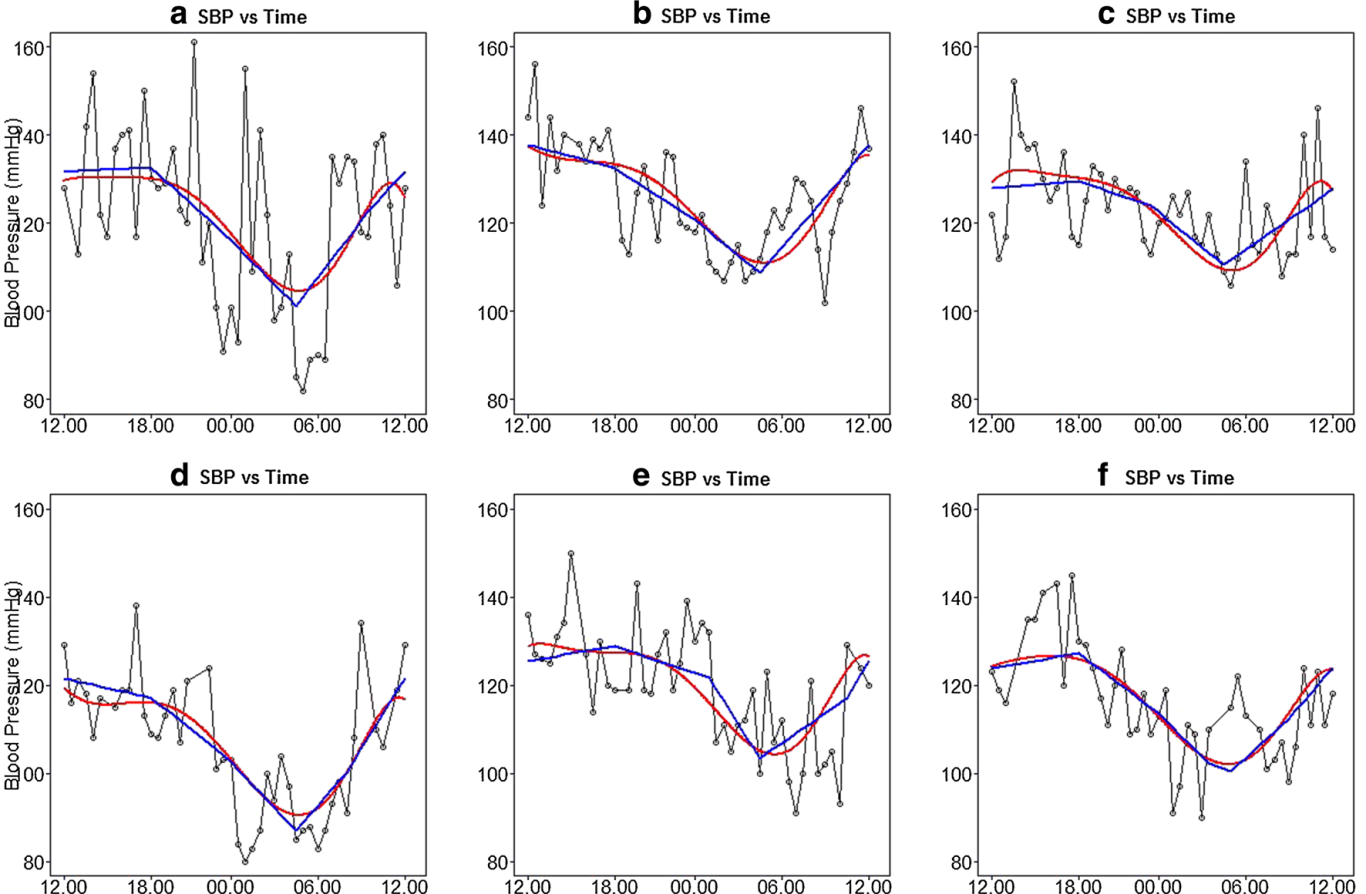
Individual BP readings along with predicted subject-specific trajectories from a linear mixed effects model as a function of time only using two different approaches; polynomials (*red line*) and piecewise linear splines (*blue lines*)

Figure 2 represents subject-specific trajectories as a function of time only from a linear mixed-effects model using both orthogonal polynomials (6th order) (red line) and piecewise linear splines (blue lines). The plots suggest that the individual curves can be adequately captured by using piecewise linear splines.

We initially included the 5 linear splines as fixed-effects. A significant improvement in fit was observed when additionally including each term individually as a random-effect, based on a LRT (all p < 0.001). As a consequence we included all the linear spline terms as random effects. To allow for temporal correlation we incorporated an AR1 structure which resulted in a significant improvement in fit (p < 0.001) (rho = 0.27). Examining the ACF plot indicated that the inclusion of an AR1 residual structure adequately accounted for the auto-correlation in the data. This unadjusted model, which only incorporates linear splines as a function of time was our base model, Table 2 (Model 1). Presented are the parameter estimates (fixed-effects, random-effects correlation matrix, the autocorrelation decay ρ along with fit criteria values). With the exception of the slope for the period from 12.00 to 18.00 [0.02 (0.04) mmHg/30 min], all slopes differed significantly from zero (all p < 0.001). This suggests that on average this is the period during the day where average SBP remains constant. The largest rise and fall in SBP occurred between wake and 12.00 (2.23 mmHg/30 min) and, between sleep and 04.00 (−1.93 mmHg/30 min) respectively. These segments correspond to the period when an individual wakes up and the period immediately after they fall asleep. The variation in slopes was lowest from 12.00 to 18.00 where the variance was 0.51. The largest variation in slopes was observed between waking and 12.00 where the variance was 2.05 which is substantially larger in comparison to the rest of the day. The model *R* ^2^ value which illustrates the proportion of variance explained by both the fixed and random factors was quite high (0.67).

**Table 2:** Various models with parameter estimates for slopes at each segment along with corresponding correlations and variances

Parameter	Model 1	Model 2	Model 3
Fixed effects	Estimate (SE)	Estimate (SE)	Estimate (SE)
BP at 12.00	134 (0.54)	119.2 (4.6)	119.3 (4.6)
Microalbuminuria	–	7.57 (1.30)*	5.79 (1.67)*
*Slope for spline time period*			
1. 12.00–18.00	0.02 (0.04)	0.03 (0.04)	0.03 (0.04)
2. 18.00–sleep	−1.00 (0.04)*	−1.00 (0.04)*	−1.01 (0.04)*
3. Sleep–04.00	−1.93 (0.05)*	−1.95 (0.06)*	−1.99 (0.06)*
4. 04.00–wake	1.69 (0.05)*	1.70 (0.05)*	1.71 (0.05)*
5. Wake–12.00	2.23 (0.07)*	2.21 (0.07)*	2.26 (0.07)*
*Microalbuminuria* *×* *spline interaction*			
1. 12.00–18.00	–	–	−0.06 (0.14)
2. 18.00–sleep	–	–	0.05 (0.13)
3. Sleep–04.00	–	–	0.37 (0.18)**
4. 04.00–wake	–	–	−0.06 (0.16)
5. Wake–12.00	–	–	−0.48 (0.22)**
*Random effects*			
Σ	223.6	199.5	200.5
	−0.23 0.51	−0.23 0.50	−0.24 0.51
	−0.23 −0.10 0.55	−0.25 −0.10 0.54	−0.25 −0.11 0.55
	−0.23 −0.45 0.03 1.39	−0.28 −0.46 0.02 1.41	−0.28 −0.44 0.02 1.40
	0.46 −0.28 −0.74 −0.05 0.66	0.47 −0.31 −0.74 −0.05 0.65	0.49 −0.33 −0.73 −0.04 0.65
	0.34 −0.06 −0.21 −0.78 0.19 2.05	0.42 −0.03 −0.22 −0.80 0.23 2.00	0.42 −0.04 −0.20 −0.81 0.24 1.97
σ	12.3	12.3	12.2
ρ	0.27	0.27	0.27
*R* ^2^	0.67	0.68	0.68
Log-likelihood	−149,608	−149,505 Model 2 versus Model 1 (p < 0.001)	−149,502 Model 3 versus Model 2 (p = 0.12)

Microalbuminuria: albumin:creatinine ratio ≥1.1 mg/mmol

Model 1: Fixed effects (5 linear splines), random effects (5 linear splines)

Model 2: Fixed effects (5 linear splines, microalbuminuria, age, sex, BMI), random effects (5 linear splines)

Model 3: Fixed effects (5 linear splines and interaction with microalbuminuria, age, sex, BMI), random effects (5 linear splines)

Random Effects matrix shown has variances on the diagonal and correlation coefficients on off-diagonals

* p < 0.001; ** p < 0.05

In subsequent models we adjusted for age, sex and BMI. We also included our variable of interest, microalbuminuria, to determine if it could help explain the larger variation in the period, wake to 12.00 (Model 2, Table 2). The residual variance, which represents the variation about an individual’s trajectory, was 12.3 mmHg. We additionally allowed the residual variance to vary between microalbuminuria groups (ratio of standard deviation of those with to without microalbuminuria was 1.09). On average, over the day, those with microalbuminuria were found to have significantly higher SBP (7.6 mmHg, 95% CI 5.0–10.1, p < 0.001). However, adjusting for age, sex, BMI and microalbuminuria had almost no effect on the model parameter estimates (except the intercept). To determine if slopes were different between groups at different times of the day we included an interaction between each linear spline and microalbuminuria (Model 3, Table 2). Although two of the interaction terms were marginally significant, a LRT suggested that including interaction terms did not improve the overall fit to the data (p = 0.12). Based on additional models (results not shown) we found no evidence that the variance of the random-effects varied with microalbuminuria. With the inclusion of age, sex, BMI and microalbuminuria we concluded that Model 2 offered the best fit to the data (Model 2 vs Model 1, p < 0.01). Residual diagnostic plots of the models (residuals vs fitted values, histogram of random-effects & residuals) showed no violation of assumptions (results not shown). The VPC plot showed the model was adequately predicting central trend and variability of SBP in the observed data, when plotted against time (see Additional file 2).

**Fig. 3:**
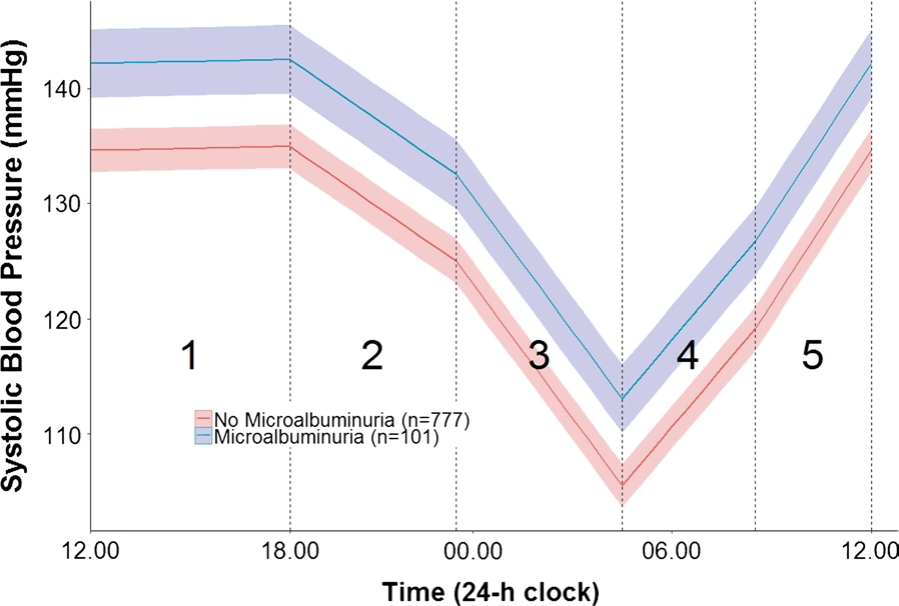
Predicted average (95% CI) piecewise linear trajectory of those with/without presence of microalbuminuria adjusted for age, sex and BMI using a linear mixed-effects model (Model 2). Each linear spline represents the rate of BP increase or decrease (slope) for that segment and has been given a corresponding number which is referred to in Table 2. For the purposes of this plot we have set the sleep and wake time knots at 23.00 and 08.00 respectively

Figure 3 represents the average piecewise linear curve along with a 95% confidence interval for those with and without the presence of microalbuminuria using Model 2. The numbers on the plot correspond to the time periods presented in Table 2. It is clear that those with microalbuminuria have a higher average SBP throughout the day. For the purposes of this plot we have set the sleep and wake time knots at 23.00 and 08.00 respectively. A similar plot using model 3 can be found in the Additional file 2. Similar findings were found for all analysis when repeated using DBP (results not shown).

## Discussion

In this large population based study we present an alternative method of modelling 24 h BP that can easily be applied to any physiological process that follows a circadian cycle. Our novel but simple approach utilising a piecewise linear random-effects model, with an adjustment to ensure that the average level is the same at the beginning and end of each 24-h period, offers a practical alternative to other methodological modelling techniques for researchers exploring circadian patterns. The flexible model has the ability to capture overall average, group and individual trajectories (in addition to being capable of examining slopes at different periods of the day).

Despite the large amounts of literature relating to BP, those specifically modelling 24 h ABPM remain sparse. Our method offers new measures of short-term BP variability as we can quantify the variation about an individual’s trajectory but it also allows examination of the variation in slopes between individuals (random-effects). Our results indicated that after adjustment for age, sex and BMI the sharpest fall in BP occurred just after an individual went to sleep and the steepest rise occurred just after waking. Although there was a significant difference on average between those with and without microalbuminuria we found there was no overall improvement in fit after including interaction effects with the spline terms. However interestingly we found that the variation after awaking, representing what is known as the morning surge was considerably larger than the other periods of the day.

It has been acknowledged there is not a generally accepted “standard” method of analysing 24-h ABPM [21]. Cosinor analysis has been highlighted as the most common approach [14–17] while fourier analysis [18], has also been implemented which are both based on the idea that any time series can be described by a series of cosine (and sine) waves of various frequencies [38]. It has been suggested that these methods impose too many restrictions on the shape of the profile and have been shown to fit real profiles poorly [39]. Wang et al. [40] suggest problems with fitting a sinusoidal function to a circadian pattern include (1) that the pattern over time may not be symmetric; that is, the peak and nadir may not be separated by 12 h and/or the amplitude and width of the peak may differ from those of the nadir, (2) sometimes there are local minimum and maximum points. Additionally Wang et al. [41] suggests that the sinusoidal function is too restrictive and “rhythms with a shape closely approximating a cosine curve are uncommon” [42]. Alternative methods have examined restricted cubic splines and more recently orthonormal polynomials [13, 21]. As we highlighted previously these approaches may model the data quite well and their curvature nature may look graphically appealing but it is difficult to understand and compare their resulting coefficients.

Piecewise regression which allows separate slopes to be fitted to observations before and after a certain period or event (knot points) has been cited as a useful tool that should be implemented more often in the context of epidemiological studies [43] but has not, to the best of our knowledge been used with ABPM data or other physiological processes that are circadian. The benefit of this method as opposed to polynomials is that the regression coefficients represent something meaningful directly without the need for further manipulation of the results—in our context, the rate of increase or decrease of BP for a certain time of day. The position of the knot points can easily be altered depending on the requirements of a specific study. For example if we were examining the effect of dialysis on BP in haemodialysis patients we could fix knot points at the time their dialysis began and at period(s) a number of hours later.

The morning is recognised as the most important period in relation to cardiovascular diseases [44] and cardiovascular events occur more frequently in this period [44–46]. In our study we found that the steepest rise (slope) occurred during the period just after waking which is in line with the literature, thus verifying that our method is capturing known features of the data. It is suggested that the abrupt steep rise in BP may explain the link between cardiovascular events and the morning period [29]. In a review of morning surge with cardiovascular risk, three different definitions of morning surge were identified, all of which simply use BP differences where they subtracted some average night value minus an average of morning BP readings [45]. We argue that our method offers a more accurate estimate, as by definition of a slope we can specifically quantify the rate of “surge”. In fact, Parati et al. argue that a method that would be capable of capturing a slope similar to one purposed by our method would provide an accurate method of estimating the morning surge [47]. Considering that morning surge has been cited as a predictor of stroke and advanced target organ damage independent of ambulatory BP and nocturnal BP [44, 46], accurately quantifying it remains an important issue, particularly when we are assessing the benefits of antihypertensive medication in their ability to reduce this steep rise. This may not only have health implications but also financial benefits. A similar argument could be put forward for the dipping effect at night which is usually quantified just as a ratio of the mean BP between night-day periods. The slope at night obtained by our approach may represent a more accurate measure but further work would be needed to explore this.

Kario argues that the perfect 24 h BP control is not limited to reducing mean BP but includes restoring disrupted circadian BP rhythms and reducing exaggerated BP variability [44]. As highlighted previously most studies examining BPV have concentrated on summary measures of variability such as SD over 24 h or separated into day and night values [5–8, 11, 12, 24]. With the use of our mixed-effects model we were are able to obtain superior measures of BPV that take into account the temporal nature of the data. We were able to quantify the variation about an individual’s trajectory but also the variation in slopes between individuals. Our work highlighted that the largest variation between individuals occurred during the morning surge period. Adjusting for age, sex and BMI did not help explain this variation. Similarly the presence of microalbuminuria had little impact on the variation. Ideally we would have preferred to explore if the variation could in part predict CV events but as data is currently only available for wave one, we have been restricted to explore a surrogate marker in microalbuminuria and have acknowledged this as a limitation. Further work is warranted to include CVD endpoints but perhaps an underlining physiological phenomenon of BP is that it is most variable in the morning possibly because this period of the day has an abrupt rise. Although some of the knots are subject-specific, others are at common fixed locations which may not represent the best position for a specific individual and this assumption is recognised as a limitation. In addition to the average plot, we have attempted to incorporate our knowledge of the underlying pattern of BP to help inform our knot positions as suggested by Howe et al. [48].

As debate remains in relation to how to correctly quantify short-term BPV [24, 49] our approach offers new alternatives that utilise the full power of ABPM that is often lost when using summary measures such as SD because it only reflects the dispersion of measurements around a single value (mean) not accounting for the order in which BP measurements were obtained [12, 50]. As argued by Bamberger et al. there are a large and increasing variety of mathematical equations available which allow us to test specific hypotheses about, and estimate parameters of, growth or change (BP) with varying purposes such as summarizing the shape of change across a sample or determining patterns in variability [51]. Despite this however, Diehl et al. suggests that summary measures and multilevel models described here are two of the most common techniques used specifically for exploring BP changes [52]. They also suggest the use of dynamic developmental models with multiple time scales as another approach to address questions related to intraindividual variability. This is quite similar to the multilevel model framework but is slightly more flexible and can include more complex scenarios. These models are characterized by time-dependent parametric changes occurring at different time scales, where each time scale defines a distinct level within a hierarchy of time scales [53]. In the context of our work this approach may be useful if we had additional data that could incorporate another time scale, such as BP over years e.g. the lifespan. The benefit from this would be that we could compare and relate BP fluctuations over 24 h to that over the lifespan. However, in this study we are only interested in 24 h variation where we only have recordings taken every 30 min. With only one time scale, we believe that the use of the traditional mixed-effects model is appropriate for our analysis. We argue that our approach, which has the ability to determine variation over specific periods of the day offers a novel measure of variability in the analysis of 24 h BP which may have benefits when attempting to determine the optimal timing of antihypertensive medication administration in future studies. Finally, as was briefly alluded to, the approach and discussion outlined is not restricted to the use of BP and can easily be implemented on any physiological process that demonstrates a circadian cycle. BP is not the only biological process where disruptions to circadian rhythms are clinical relevant. Wang et al. found that those with Cushing syndrome exhibited no circadian rhythm of cortisol, while those with depression showed a dampened rhythm compared to the normal group [41]. Liu et al. found that larger short-term fluctuations in intraocular pressure are more common in glaucoma [54]. Similar to the morning BP surge, it was found that intraocular pressure was higher in the morning and more prevalent in those with glaucoma. This suggests that our approach may be beneficial to the exploration of other biological rhythms that have similar features to that of BP.

## Conclusion

This study has introduced a novel but practical method for the analysis of ABPM data. Based on our work circadian BP patterns can be modelled using a mixed-effects model with piecewise linear splines. The main advantage of our method compared to other approaches is that the resulting regression coefficients have direct interpretation. We can determine the rate of increase or decrease at different periods of the day. In addition we can determine alternative measures of variability compared to classical BPV indices. Future research in this area should focus on the association between the measures obtained from this method to stronger clinical outcomes.

## Additional files

**Additional file 1.** Example of statistical code in R.

**Additional file 2.** VPC plot and predicted average piecewise linear trajectory for Model 3 (interaction model).

## Abbreviations

ABPM: ambulatory blood pressure monitoring
BP: blood pressure
BPV: blood pressure variability
SD: standard deviation
ACR: albumin:creatinine ratio
REML: restricted maximum likelihood estimation
EBLUP: Empirical Best Linear Unbiased Predictors
VPC: visual predictive check
AR(1): autoregressive
