# Supplementary material for: Exploring diurnal variation using piecewise linear splines: an example using blood pressure
Source: Emerg Themes Epidemiol. 2017 Feb 2;14:1. doi: 10.1186/s12982-017-0055-5 (PMC5290604; doi:10.1186/s12982-017-0055-5)
Supplement: Supplementary file 1 — Additional file 1. Example of statistical code in R. [file 12982_2017_55_MOESM1_ESM.pdf]

```
##########################################################################
####Sample code: Exploring diurnal variation using piecewise linear################
####splines: an example using blood pressure####################################
##########################################################################
rm(list = ls()) #clear

library(foreign)   #imports stata file
library(nlme)
library(multcomp)

getwd()
setwd("C:/Users/JM")
w1 <- read.dta("w1.dta")
w1<-w1[order(w1$id, w1$newtime),]

####newtime 0-47 represents 24h clock
####newsleep represents subject-specific sleep time
####newwawke represents subject-specific wake time

####create individual splines with restriction BP on the average is cyclical ####
####create 5 splines - t12pm,t6pm,tsleep,t4am,twake
w1$t12pm<-ifelse(w1$newtime<=12, w1$newtime, 12)
w1$t6pm <- ifelse(w1$newtime<=12, 0, ifelse((w1$newtime>12) & (w1$newtime<=w1$newsleep),
(w1$newtime-12), (w1$newsleep-12)))
w1$tsleep<-ifelse(w1$newtime<=w1$newsleep, 0, ifelse((w1$newtime>w1$newsleep) &
(w1$newtime<=32), (w1$newtime-w1$newsleep), (32-(w1$newsleep))))
w1$t4am<- ifelse(w1$newtime<=32 ,0, ifelse((w1$newtime>32) & (w1$newtime<=w1$newwake),
(w1$newtime-32), (w1$newwake-32)))
w1$twake<-ifelse(w1$newtime<=w1$newwake, 0, ifelse((w1$newtime>w1$newwake) &
(w1$newtime<=47), (w1$newtime-w1$newwake), (47-(w1$newwake))))

####code introducing restriction ensuring pattern is periodic
w1$s2time<-w1$newsleep-12
w1$s3time<-32-w1$newsleep
w1$s4time<-w1$newwake-32
w1$s5time<-47-w1$newwake

#final splines - s2,s3,s4,s5
w1$s2<-w1$t6pm-((w1$s2time/12)*w1$t12pm)
w1$s3<-w1$tsleep-((w1$s3time/12)*w1$t12pm)
w1$s4<-w1$t4am-((w1$s4time/12)*w1$t12pm)
w1$s5<-w1$twake-((w1$s5time/12)*w1$t12pm)
##########################################################


####Unadjusted Model 1 ####
m1r<- lme(sbp~ s2 + s3 + s4 + s5 ,
        random = ~ s2 + s3 + s4 + s5 | id ,method="REML",
        data=w1,control = lmeControl(msMaxIter=1000,opt = "optim",msVerbose=T),
        na.action="na.omit",correlation = corAR1(form=~1|id))
summary(mr1)
```

```
####variance-covariance matrix - random effects
getVarCov(m1r)
####need to go back to work out s1 estimate using linear combinations using glht command
####s1 refers to first spline rewritten
summary(glht(m1r, linfct = c("(s2*10+s3*10+s4*8+s5*7)/-12=0")))   #0.0158 se=0.037


##############################################################################
##############################################################################
```
