# Supplementary material for: Exploring diurnal variation using piecewise linear splines: an example using blood pressure
Source: Emerg Themes Epidemiol. 2017 Feb 2;14:1. doi: 10.1186/s12982-017-0055-5 (PMC5290604; doi:10.1186/s12982-017-0055-5)
Supplement: Supplementary file 2 — Additional file 2. VPC plot and predicted average piecewise linear trajectory for Model 3 (interaction model). [file 12982_2017_55_MOESM2_ESM.docx]

**Supplementary content**


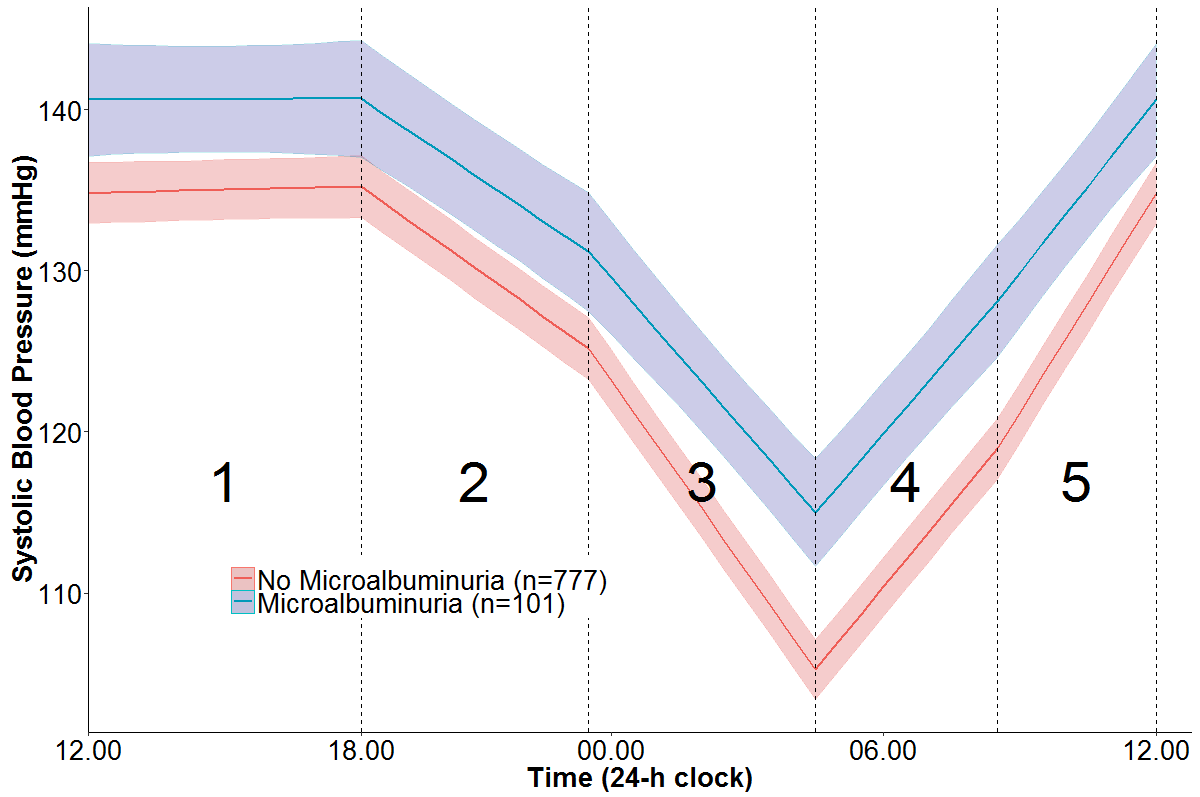


Predicted average (95% CI) piecewise linear trajectory of those with/without presence of microalbuminuria adjusted for age, sex, BMI and including an interaction of the spline terms with microalbuminuria using a linear mixed-effects model (Model 3). Each linear spline represents the rate of BP increase or decrease (slope) for that segment and has been given a corresponding number which is referred to in Table 2.


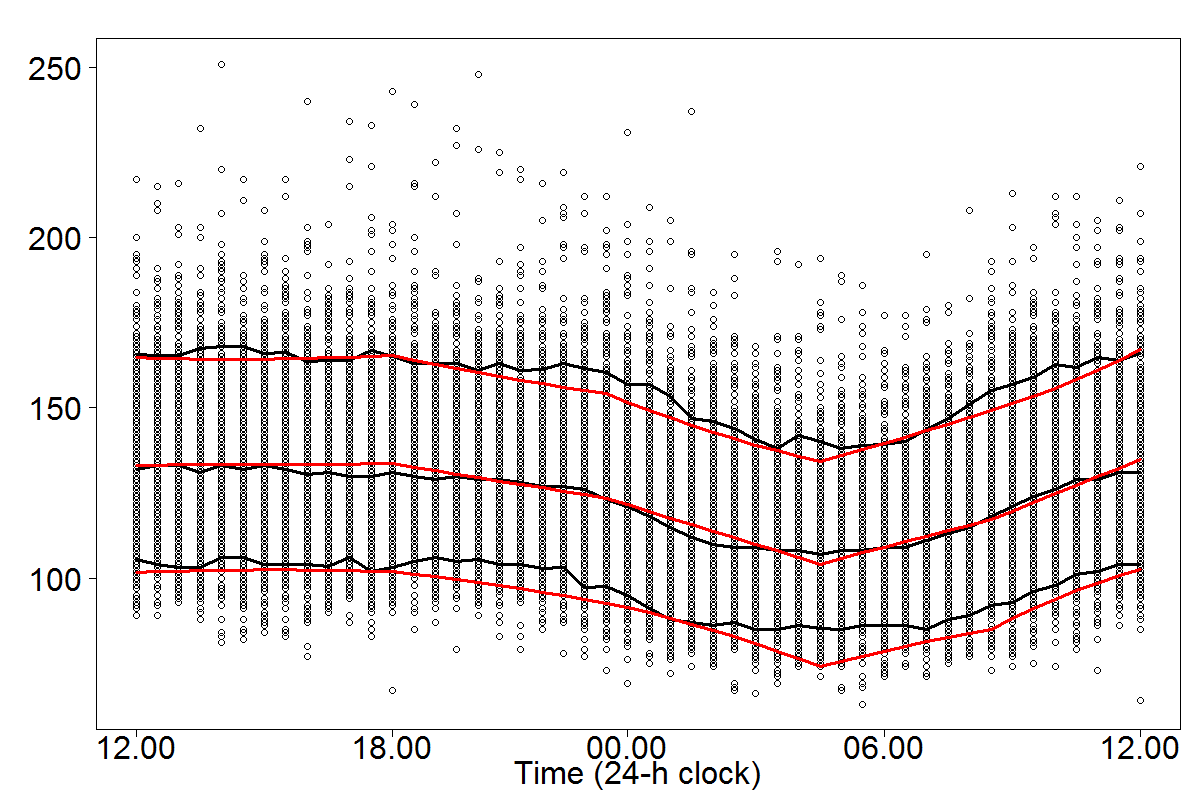


VPC (visual predictive check)plot. Black lines represent median of observed data with 90% interquantile range of observations. Red line is predicted mean along with 90% prediction interval.
